# Beyond conventional imaging: A systematic review and meta-analysis assessing the impact of computed tomography perfusion on ischemic stroke outcomes in the late window

**DOI:** 10.1177/17474930241292915

**Published:** 2024-11-10

**Authors:** Salah Elsherif, Brittney Legere, Ahmed Mohamed, Razan Saqqur, Nida Fatima, Maher Saqqur, Ashfaq Shuaib

**Affiliations:** 1 Faculty of Health Sciences, Queen’s University, Kingston, ON, Canada; 2 Department of Applied Sciences, University of Guelph, Guelph, ON, Canada; 3 Schulich School of Medicine & Dentistry, University of Western Ontario, London, ON, Canada; 4 Department of Health, University of Waterloo, Waterloo, ON, Canada; 5 Department of Neurosurgery, The University of Texas Southwestern Medical Center, Dallas, TX, USA; 6 Department of Neurology; Trillium Health Partners, University of Toronto, Toronto, ON, Canada; 7 Department of Neurology, University of Alberta, Edmonton, AB, Canada

**Keywords:** CT scan, CT perfusion ischemic stroke, stroke

## Abstract

**Background:**

Non-contrast cranial computed tomography (NCCT) and CT angiogram (CTA) have become essential for endovascular treatment (EVT) in acute stroke. Patient selection may improve when CT perfusion (CTP) imaging is also added for patient selection. We aimed to analyze the effects of implementing CTP in acute ischemic stroke (AIS) patients’ treatment to assess whether stroke outcomes differ in the late window.

**Methods:**

We searched the PubMed, Embase, and Web of Sciences databases to obtain articles related to CTA and CTP in EVT. Collected patient data were split into two groups: the CTP and control (NCCT + CTA) cohorts. Primary outcomes evaluated were modified Rankin Scale (mRS) scores, symptomatic intracranial hemorrhages (sICHs), mortality, and successful recanalization.

**Results:**

There were 14 studies with 5809 total patients in the final analysis: 2602 received CTP and 3202 were in the control group. CTP/CTA patients showed significantly lower rates of 90-day stroke-related mortality (odds ratio (OR) = 0.72, 95% confidence interval (CI) = 0.60–0.87, *p* < 0.01) and significantly higher successful recanalization (OR = 1.42, 95% CI = 1.06–1.94, *p* < 0.01) compared with CTA-only patients. Analysis of other outcomes including functional independence (mRS = 0–2), critical times, and intracranial hemorrhages was non-significant (*p* > 0.05).

**Conclusion:**

The study highlights the usefulness of CTP-guided therapy as a supplementary tool in EVT selection in the late window. Although the addition of CTP resulted in lower mortality, the favorable outcomes did not improve. Further evidence is required to establish a clearer understanding of the potential advantages or limitations of incorporating CTP in stroke imaging.

## Introduction

Successful endovascular treatment (EVT) of acute ischemic stroke (AIS) depends on proper patient section and early appropriate management. Imaging methods commonly utilized include non-contrast cranial computed tomography (NCCT), CT angiography (CTA), and CT perfusion (CTP). Although the original early time window trials of EVT in less than 6 h did not require CTP for patient selection,^1^ more recent trials especially in the prolonged time window^2,3^ or in patients with large-core stroke^4,5^ utilized CTP for patient selection. Some recent large-core trials used the Alberta Stroke Program Early CT Score (ASPECTS) score on NCCT and CTA for selection for EVT,^6^ questioning the requirement of CTP imaging in patient selection. CTP imaging gathers additional data regarding lesion location, collateral status, and severity of damage to surrounding brain tissue that is not evident on NCCT. It is, however, unclear whether this additional CTP data can directly modify patient outcomes and selection for endovascular therapy (EVT).

An important advantage of CTP imaging is that it may provide quantitative perfusion metrics and means to distinguish infarct core and tissue at risk.^7^ CTP can improve the likelihood of diagnosing smaller distal occlusions.^8^ It is also a very helpful tool when patients with a suspected stroke are being investigated for potential EVT. The likelihood of an intracranial large vessel occlusion in the presence of a normal CTP is very uncommon.^9^ CTP imaging is, however, not widely available and requires additional contrast use. Kim et al.^10^ found that only 3% of AIS patients utilized CTP as their primary imaging tool when retrospectively analyzing Medicare data sets in Texas. Furthermore, large regions of the state needed immediate access to CTP, and hospitals that offered CTP were mostly located within urban areas.^10^ There is, therefore, renewed interest in evaluating EVT of acute stroke in the prolonged time window beyond 6 h with imaging limited to NCCT and CTA.

A recent systematic review by Kobeissi et al.^11^ found that the addition of CTP imaging resulted in lower rates of mortality and higher rates of reperfusion. Some recent trials including TENSION argue that using NCCT and CTA modalities for patient selection yielded similar clinical benefits to the more sophisticated, time-consuming, and complicated CTP.^12^ Considering these conflicting results, the challenge remains as to whether CTP imaging offers additional value in patient selection for EVT, especially in large-core or late-window patients.

This meta-analysis aimed to analyze and include recently published data containing a larger patient cohort in our systematic review. Our main objective was to evaluate whether CTP plays a role in proper patient selection and better outcomes in an acute stroke setting in the late window for EVT treatment. In addition, we aimed to assess the functional independence and clinical outcomes in AIS patients to determine the safety of conventional imaging standards (NCCT and CTA) and whether the integration of CTP alters these outcomes.

## Methods

### Data search strategy

The Preferred Reporting Items for Systematic Review and Meta-analysis (PRISMA) was utilized to conduct the literature search for our study.^13^ Three authors (S.E., B.L., and A.M.) thoroughly reviewed publications on the following databases to select studies for our meta-analysis: PubMed, EMBASE, Cochrane Library, and Google Scholar. The studies included were published between October 2015 and October 2023. The searched MeSH terms were “computed tomography perfusion,” “brain imaging,” “ischemic stroke imaging,” “ischemic stroke treatment,” “non-contrast computed tomography,” and “computed tomography angiography.”

### Data extraction and outcome measures

The data extraction was completed by three authors (S.E., B.L., and A.M.) following PRISMA guidelines.^13^ Discussion between the authors was used to resolve any conflicts. Extracted data included (1) baseline characteristics (location, total patients, mean age, and distribution of patients in CTP and control cohort), (2) clinical outcomes (modified Rankin Scale (mRS) scores at 90-day post-operation, symptomatic intracranial hemorrhage (sICH), mortality, and successful recanalization), and (3) critical times (door-to-needle times).

Investigation of primary outcomes included the analysis of clinical outcomes defined by mRS scores at 90-day post-operation. mRS scores were dichotomized and split into two groups: good outcome (0–2) and poor outcome (3–6). Mortality was defined as a mRS value of 6. Secondary outcome analysis addressed stroke-related events, mortality, and critical times.

### Inclusion and exclusion criteria

We included randomized controlled trials (RCTs), as well as retrospective or prospective studies that compared AIS patients receiving conventional imaging (NCCT + CTA) and CTP to control patients receiving conventional imaging protocols only. Studies that assessed this association in the early window were excluded (<6 h). All other studies that did not include both cohorts and assessed comparison of other types of imaging were excluded as these may create confounding variables. Moreover, case–control studies, case series, and case reports were excluded.

### Statistical analysis

Mean difference (MD) and standard deviation were utilized to compare continuous data while the odds ratio (OR) was utilized to compare dichotomous data. Data were presented as either MD or OR with a 95% confidence interval (CI). Rev Manager 5.3 was used for data comparison of the included studies. For heterogeneity, a fixed model was used if *I*^2^ was <50%, and a random effect model was used if *I*^2^ was >50%. A *p* value of <0.05 was deemed statistically significant.

### Risk of bias across studies

High heterogeneity was evaluated using a funnel plot which indicated an asymmetrical distribution. The removal of small sample-sized cohorts significantly decreased the heterogeneity when analyzed from a funnel plot forming an asymmetrical distribution.

## Results

### Study selection

**Figure 1.**
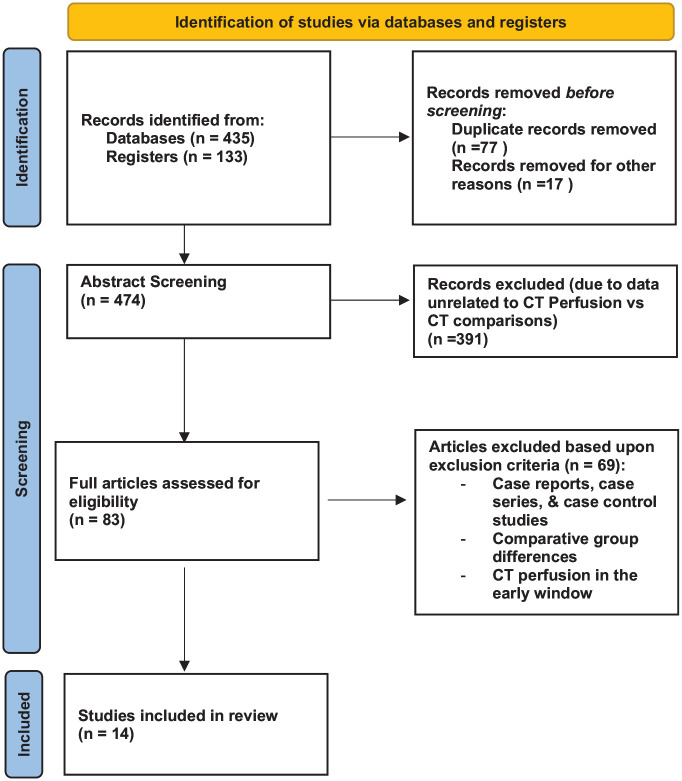
PRISMA guidelines.

Articles were reviewed according to PRISMA guidelines (Figure 1).^13^ A total of 14 studies with 5809 patients were included in the final meta-analysis. Out of these patients, 2602 were included in the CTP-triaged cohort while 3202 were included in the control cohort, receiving conventional imaging techniques.

Seven studies were retrospective analyses of patient data, while seven studies included prospective collection and analysis of data.^2,3,12,14–17^ These studies were conducted in the United States^3,18^ (*n* = 2), Canada^19^ (*n* = 1), the United Kingdom^17^ (*n* = 1), Australia and New Zealand^14,20^ (*n* = 2), Germany^21^ (*n* = 1), Spain^12^ (*n* = 1), France^15^ (*n* = 1), and South Korea^22^ (*n* = 1). Other studies were conducted internationally across multiple comprehensive stroke centers in multiple regions.^2,16,23^ Only three were single-center studies that highlighted patient data collected in a single site.^12,20,24^

### Patient characteristics

The mean ages for the CTP and control cohort were 68.79 ± 4.17 and 69.37 ± 4.1 years, respectively. Patient baseline data showed no significant difference in onset/last seen well (LSW)-to-groin puncture time (CTP mean ± SD = 582.6 ± 166.9 min vs control mean ± SD = 503.0 ± 171.1; *p* = 0.32). Table 1 reports other baseline characteristics, including the National Institutes of Health Stroke Scale (NIHSS) scores and internal carotid artery (ICA) occlusions. The primary outcomes included mRS and 90-day stroke-related mortality, and secondary outcomes reported sICH and successful recanalization (Table 2).

**Table 1.** Baseline Characteristics.

Study	Study type	Study design	Ethnicity/Location	Total patients	Primary/Comprehensive stroke center	Mean age (CTP)	Mean age (control)	Perfusion group (NCCT + CTA + CTP)	Standard group (NCCT + CTA)	Male sex—No. (%)		Door-to-groin puncture (min)		Onset/LSW-to-groin puncture (min) (late window)		NIHSS		Rate of thrombolysis—No. of patients (%)		ICA		M1 MCA		M2 MCA	
					Control	CTP	Control	CTP	Control	CTP	Control	CTP	Control	CTP	Control	CTP	Control	CTP	Control	CTP	Control	CTP	Control	CTP	Control
Nogueira et al., 2018	Prospective	RCT	International	206	Multicenter	69.4 ± 14.1	70.7 ± 13.2	107	99	42 (39)	51 (52)	NA	NA	NA	NA	17 (13–21)	17 (14–21)	5 (5)	13 (13)	22 (21)	NA	83 (78)	NA	2 (2)	NA
Ma et al., 2019	Prospective	RCT	Australia, New Zealand, Taiwan, Finland	225	Multicenter	73.7 ± 12.7	71.0 ± 12.7	113	112	59 (52)	66 (59)	124 (81–179)	127 (87–171)	NA	NA	12 (8–17)	10 (6–17)	NA	NA	NA	NA	NA	NA	NA	NA
Albers et al., 2018	Prospective	RCT	USA	182	Multicenter	70 (59–79)^a^	71 (59–80) ^a^	92	90	46 (50)	44 (49)	NA	NA	NA	NA	16 (10–20) ^a^	16 (12–21) ^a^	10 (11)	8 (9)	32/92 (35)	NA	60/92 (65)	NA	NA	NA
Bouslama et al., 2017	Retrospective	Registry-based	USA	602	Multicenter	63.9 ± 15.2	67.8 ± 14.6	365	237	184 (50)	132 (56)	NA	NA	365 (244–587)	284 (208–425)	18 (13–22)	19 (15–22)	145 (40)	111 (47)	NA	NA	NA	NA	NA	NA
Furlanis et al., 2019	Retrospecitve	Registry-based	Italy	149	Single center	73 ± 13	79 ± 10	75 (50)	74 (50)	39 (52)	30 (41)	NA	NA	527 (383–702)^b^	684 (555–848) ^b^	6 (4–13)	7 (3–16)	NA	NA	NA	NA	NA	NA	NA	NA
Medina-Rodriguez et al., 2020	Prospective	Observational	Spain	657	Comprehensive	72.8 ± 11.3	69.3 ± 13.5	53 (8)	604 (92)	29 (55)	288 (48)	43	25	538 ± 76	155 ± 5	9.79 ± 2.5	13.1 ± 0.57	26 (72)	252 (52)	NA	NA	NA	NA	NA	NA
Menjot de Champfleur et al., 2017	Prospective	RCT	France	173	Multicenter	68 (59–75)^a^	71 (64–77)^a^	139 (80)	34 (20)	76 (55)	9 (27)	NA	NA	NA	NA	17 (13–19)	17 (13–21)	NA	NA	17 (13)	7 (23)	101 (76)	22 (71)	15 (11)	2 (7)
Garcia-Esperon et al., 2022	Retrospective	Registry-based	Australia and New Zealand	131	Comprehensive	65 (58–74)^a^	64 (52–70)^a^	52 (61)	79 (46)	24 (46)	48 (61)	NA	NA	NA	NA	16 (10–21)^a^	16 (12–22)^a^	11 (21)	9 (11)	6 (12)	10 (13)	30 (58)	38 (49)	8 (15)	7 (9)
Nguyen et al., 2021	Retrospective	Observational	International	1286	Multicenter	69 (58–80)^a^	71 (58–81)^a^	752 (58)	534 (42)	346 (46)	261 (49)	NA	NA	678 (504–912)	624 (468–864)	16 (11–19)	17 (13–21)	91 (12)	126 (24)	162 (22)	161 (30)	430 (57)	300 (56)	160 (21)	73 (14)
Nogueira et al., 2021	Prospective	Registry-based	International	247	Multicenter	78.5 ± 14.5	70.3 ± 13.6	180 (73)	67 (27)	74 (41)	39 (58)	NA	NA	204 (150–270)	186 (144–246)	15 (11–20)	16 (12–20)	48 (27)	16 (24)	38 (21)	13 (19)	107 (59)	45 (67)	35 (19)	9 (13)
Porto et al., 2022	Retrospective	Registry-based	Canada	699	Multicenter	70 (60–81)^a^	68 (57–78)^a^	280 (40)	419 (60)	134 (48)	193 (46)	NA	NA	742 (490–1020)	662 (466–931)	14 (8–19)	15 (10–19)	40 (14)	59 (14)	97 (35)	140 (33)	137 (49)	178 (43)	46 (16)	101 (24)
Dhillon et al., 2022	Prospective	Registry-based	United Kingdom	1046	Multicenter	NA	NA	378 (36)	668 (64)	188 (50)	366 (55)	NA	NA	672 ± 251	619 ± 548	16 (10–21)	16 (9–20)	104 (28)	226 (34)	NA	NA	NA	NA	NA	NA
Kim et al., 2019	Retrospective	Registry-based	Korea	50	Multicenter	69.9 ± 12.8	75.0 ± 3.5	47 (94)	3 (6)	26 (55)	3 (100)	NA	NA	766 ± 288	596 ± 79	14 (6–22)	13 (11–19)	NA	NA	NA	NA	NA	NA	NA	NA
Herzberg et al., 2021	Retrospective	Registry-based	Germany	156	Multicenter	75.56 ± 13.08	72.29 (12.66)	27 (17)	129 (83)	14 (52)	55 (43)	NA	NA	NA	NA	16 (13.5–18)	16 (13–20)	10 (37)	57 (44)	10 (37)	47 (35)	20 (74)	95 (73.6)	NA	NA

a Median.

b LSW to admission time.

**Table 2.** Study Outcomes.

Study	Patients with functional independence (mRS = 0–2) at 90 days (%)		Rate of symptomatic intracranial hemorrhage		90-day stroke-related mortality		Successful recanalization		Rate of thrombolysis	
	CTP	Control	CTP	Control	CTP	Control	CTP	Control	CTP	Control
Nogueira et al., 2018	52/107 (49)	NA	6/107 (6)	NA	17/107 (16)	NA	82/107 (77)	NA	NA	NA
Ma et al., 2019	56/113 (50)	NA	7/113 (6.2)	NA	13/113 (11.5)	NA	72/107 (67.3)	NA	NA	NA
Albers et al., 2018	41/92 (45)	NA	6/92 (7)	NA	13/92 (14)	NA	69/91 (76)	NA	10 (11)	NA
Bouslama et al., 2017	175/365 (53)	86/237 (40)	NA	NA	55/365 (17)	57/237 (27)	351/365 (96)	212/237 (90)	145/365 (40.1)	111/237 (46.8)
Furlanis et al., 2019	29/75 (39)	40/74 (54)	NA	1/74 (2)	6/75 (8)	5/74 (7)	NA	NA	NA	NA
Medina-Rodriguez et al., 2020	36/53 (72)	317/604 (53)	2/53 (3.8)	22/604 (3.8)	4/53 (7.5)	68/604 (12)	NA	NA	NA	NA
Menjot de Champfleur et al., 2017	71/139 (51)	17/34 (50)	NA	NA	NA	NA	97/139 (70)	21/34 (61)	33.1	NA
Garcia-Esperon et al., 2022	30/52 (58)	32/79 (41)	NA	NA	NA	NA	NA	NA	11/52 (29.2)	9/79 (11.4)
Nguyen et al., 2021	333/752 (44)	220/534 (41)	43/752 (5.8)	42/534 (8.1)	159/752 (21)	125/534 (23)	670/752 (89)	474/534 (89)	91/752 (12)	126/534 (24)
Nogueira et al., 2021	99/180 (55)	41/67 (61)	1/180 (0.56)	1/67 (1.5)	20/180 (11)	6/67 (9)	169/180 (94)	64/67 (96)	49/180 (27)	16/67 (24)
Porto et al., 2022	83/280 (35)	92/419 (38)	37/280 (14)	34/419 (9)	38/280 (21)	78/419 (27)	246/280 (89)	351/419 (84)	40/280 (14)	59/419 (14)
Dhillon et al., 2022	67/122 (55)	112/207 (54)	10/298 (3.4)	20/431 (4.6)	39/378 (10)	98/668 (15)	318/378 (84)	523/668 (78)	104/378 (28)	226/668 (34)
Kim et al., 2019	29/47 (61.7)	2/3 (66.7)	NA	NA	1/47 (2.1)	0/3 (0)	42/47 (89.4)	3/3 (100)	NA	NA
Herzberg et al., 2021	5/27 (19)	36/129 (28)	9/27 (33)	21/129 (16)	8/27 (30)	43/129 (33)	24/27 (89)	101/129 (78)	10/27 (37)	57/129 (44)

### Primary outcomes

#### Favorable functional outcome mRS 0–2 (90 days)

**Figure 2.**
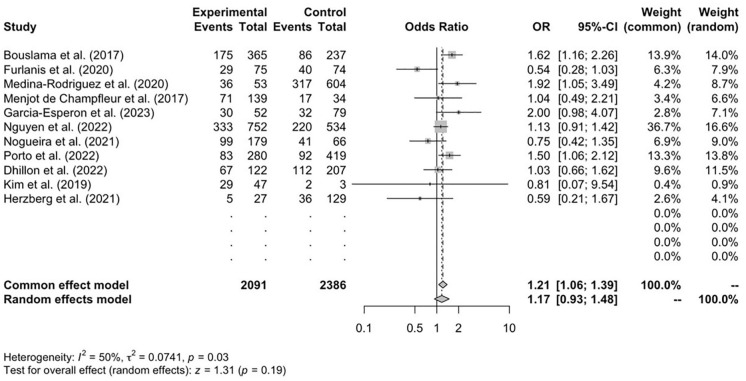
Functional independence at 90 days (mRS = 0–2), *n* = 11.

Eleven studies^12,15–24^ reported data on favorable functional outcomes (mRS = 0–2) at 90 days for both the CTP and control cohorts. Rates of favorable outcomes between the cohort that had CTP imaging and the control cohort showed no significant difference (OR = 1.17; 95% CI = 0.93, 1.48; *p* = 0.19) (Figure 2).

### Mortality

**Figure 3.**
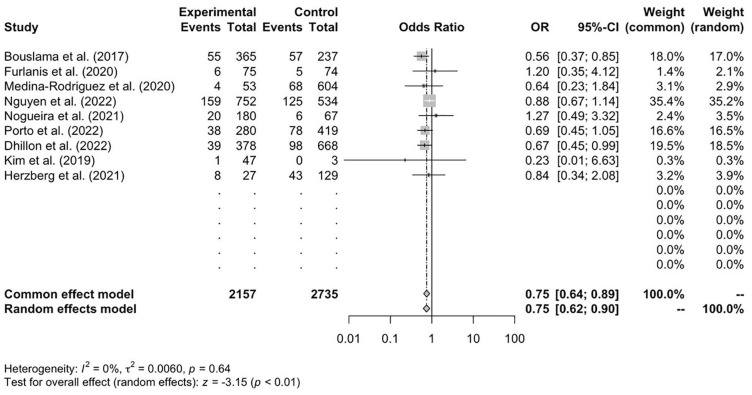
Mortality at 90 days, *n* = 9.

Nine studies^12,16–19,21–24^ reported 90-day mortality data for the CTP and control cohorts. There was a higher rate of mortality in the control cohort compared with the CTP-assessed cohort. The CTP group reported a significantly lower mortality rate at 90 days, and mean reported value was 14.2 % (*n* = 330) compared with 16.4% (*n* = 480) in the control (OR = 0.75; 95% CI = 0.62–0.90; *p* < 0.01) (Figure 3).

### Secondary outcomes

#### Successful recanalization and sICH

**Figure 4.**
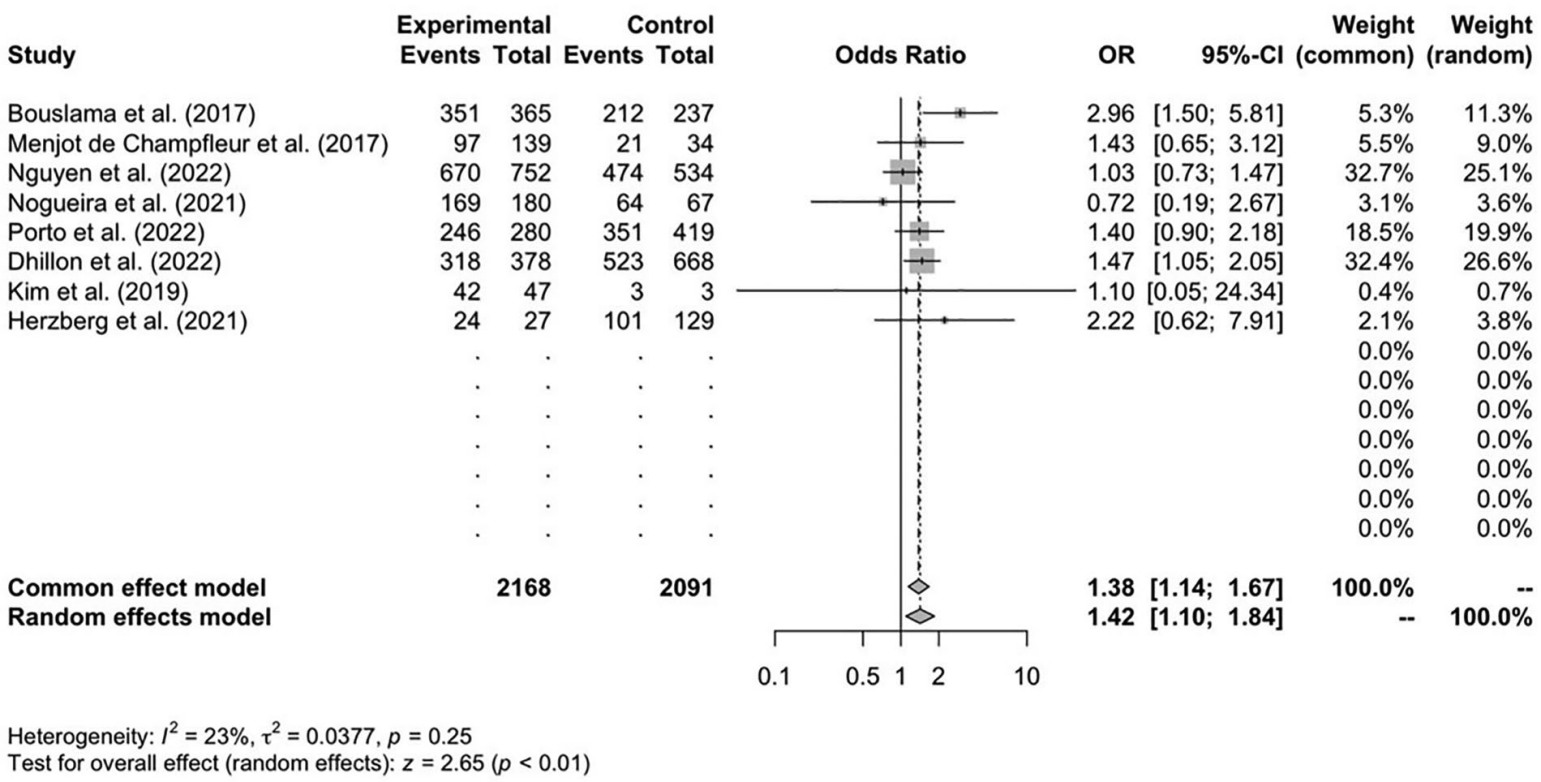
Successful recanalization at 90 days, *n* = 8.

Eight^15–19,21–23^ of 14 studies reported data on successful recanalization in both cohorts. The CTP-triaged cohort revealed a 1.43-fold higher likelihood of successful recanalization, defined by a thrombolysis in c﻿erebral infarction (TICI) score of 2b or 3 (1.42; 95% CI = 1.10–1.84; *p* < 0.01) (Figure 4). When conducting a sub-group analysis for the CTP cohort, retrospective studies exhibited higher successful recanalization rates compared with prospective studies (*p* = 0.05).

**Figure 5.**
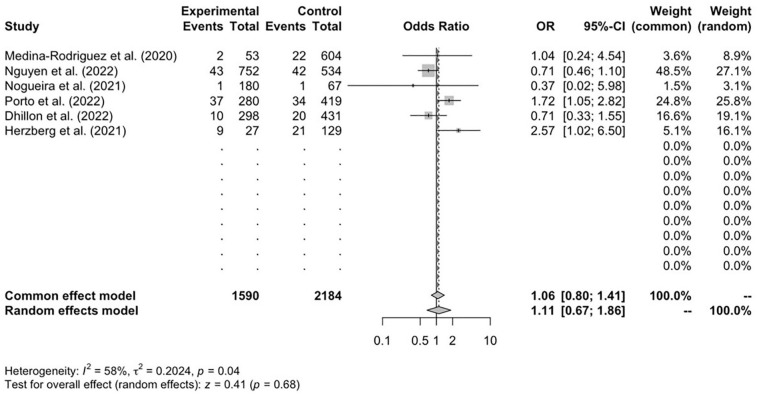
Symptomatic intracranial hemorrhage (sICH) at 90 days, *n* = 6.

Alternatively, the CTP-assessed cohort revealed a significantly lower successful recanalization percentage mean of 84.1% (*n* = 1917) relative to an 87.7% mean (*n* = 1749) for the control cohort. Six studies^12,16,17,19,21,23^ reported data on sICH within both groups. Results revealed no difference in sICH values between CTP (*n* = 957) and control (*n* = 995) cohorts (1.11; 95% CI = 0.67–1.86; *p* = 0.68) (Figure 5). After conducting a sub-group analysis, retrospective studies also demonstrated significantly higher sICH rates relative to prospective studies (*p* = 0.02).

## Discussion

Our systematic review and meta-analysis showed that the addition of CTP imaging in comparison with NCCT and CTA results in a significant decrease in mortality. In addition, the CTP-triaged cohort reported significantly higher rates of successful recanalization. While our results were in line with another systematic review,^9^ our analysis included 10^2,3,12,14,15,18,20–22,24^ additional studies with large-core stroke populations. Second, CTP imaging did not impact functional outcomes including mRS at 90 days, rates of thrombectomy, and early risk of sICH. The lack of additional benefit in the 90-day outcome with the additional information obtained with the use of CTP is, therefore, surprising. CTP especially when used with AI software can provide reliable information on the volume of the ischemic core and the size of the penumbra. In addition, the hypoperfusion index (HI) allows for a valuable approximation of the collateral status.^25^ A recent analysis of the INSPIRE registry showed that CTP was a more reliable indicator of 90-day outcome compared with ASPECTS score.^26^ Another recent retrospective analysis of CTP in acute stroke suggested that the size of the penumbra may not influence outcome.^27^ It is important to note that although CTP findings may have an impact on treatment selection, there are other variables that can impact these treatment decisions as well. For example, NIHSS, ASPECTS, pre-stroke mRS scores, and other patient-specific factors may influence treatment decisions and planning. Majority of conventional imaging modalities for AIS patients included NCCT and CTA imaging. NCCT is utilized within the specific time frame of 4.5 h to exclude intracerebral hemorrhage and select patients for intravenous therapy (IvTPA).^28^ CTA imaging is utilized to confirm arterial occlusion and to understand cerebral vasculature health and the effects of collaterals on the occluded region of the brain.^28^ These two imaging modalities are essential for the identification and selection of patients for EVT. The inclusion of additional imaging, such as magnetic resonance imaging (MRI), has been proven to improve diagnostic accuracy and better predict better patient outcomes.^29^ Our study sought to exclusively evaluate different CT imaging modalities in the extended time window, and we, therefore, excluded comparisons with MRI and other imaging forms. As guidelines do not recommend the routine use of CTP in patient selection for patients presenting in under 4.5 h, we restricted our evaluation to patients treated in the delayed time window.

Recently, the DAWN,^2^ DEFUSE 3,^3^ and EXTEND-IA^30^ studies have all revealed the effectiveness of EVT in AIS patients selected using AI-assisted perfusion imaging. CTP imaging provided clinicians with tissue perfusion patterns, and the additional information defining the size of the ischemic core and penumbra was likely helpful in the successful outcomes of these delayed treatment studies.

Our results support that the inclusion of CTP in patients presenting in the late window may benefit from the additional imaging by reducing mortality at 90 days and, in addition, significantly increasing the chances of successful recanalization. Considering the similar baseline age (*p* = 0.93), NIHSS scores (*p* = 0.76), and onset to groin puncture times (*p* = 0.32) between both the CTP and non-CTP groups shown in Table 3, this further highlights the effectiveness of CTP in selecting patients for treatment. Whereas we were unable to show that this will result in a better outcome, there is evidence that improved recanalization rates were associated with increased salvaged penumbra volume and better functional independence.^31^ With a larger cohort analyzed, our study provides a continuation of previous work that has evaluated the efficacy of perfusion imaging implementation in AIS patient treatment. Katyal et al. conducted a meta-analysis of 22 studies with a total of 5623 patients to evaluate the efficacy of CTP-guided therapy in AIS patients.^32^ The study revealed that CTP-guided therapy provided benefits both within and outside the clinically recommended window for reperfusion. Furthermore, although only included five studies in their review, Kobeissi et al. (2023) revealed similar results to our study with significantly higher rates of successful recanalization and significantly lower rates of mortality in patients imaged with CTP compared with NCCT.^11^ The results of these studies suggest that perfusion imaging may be used as a supplementary tool to assist with patient selection in the late window for reperfusion therapy, especially for wake-up stroke individuals. While our results suggest that selection with CTP significantly reduces mortality, this may be due to other confounding variables. Previous research has demonstrated that patients with large-core infarctions or no penumbral patterns may not receive EVT. Ischemic stroke patients assessed with CTP may then be excluded from treatment due to the over triaging and potential bias in the selection of these patients. For example, Bouslama et al. (2017) reported lower rates of IvTPA treatment within the CTP group compared with the NCCT group (40.1% vs 46.8%; *p* = 0.109). Although this difference was non-significant, the study found that CTP implementation significantly increased the odds of good outcomes (mRS = 0–2) for patients (OR = 1.72, 95% CI = 1.10–2.67, *p* = 0.017). Another study that demonstrated this pattern was Nguyen et al. (2022), which revealed that 12.1% of patients who had received CTP were administered IvTPA. In contrast, 23.6% of patients who underwent conventional imaging received IvTPA. The significantly lower treatment rates for CTP patients reiterate the point that CTP assessment may act as a confounding variable and affect patient outcomes by over-selecting patients for treatment. Therefore, while our review suggests lower mortality with CTP imaging implementation, it is important to interpret these results with caution as the lower mortality may be due to over-selection of patients by CTP, potentially impacting outcomes for patients.

**Table 3.** Baseline Group Comparisons.

	Perfusion group (NCCT + CTA + CTP)	Control group (NCCT + CTA)	*p*
Total patients	2660	3149	N/A
Age	68.8 ± 4.17	69.4 ± 4.1	0.93
Gender (% males)	49.4 ± 4.96	53.14 ± 16.1	0.41
NIHSS	14.5 ± 3.3	14.86 ± 3.2	0.76
Rate of thrombolysis (%)	26.7 ± 19.6	27.2 ± 16.1	0.95
Onset-to-groin puncture (min)	582.6 ± 166.9	503.0 ± 171.1	0.32

Although there is consensus among studies on the direct advantages of CTP, including higher sensitivity and easier patient selection for EVT, the routine implementation of this technology can only be justified with overall improved patient outcomes. Lopez-Rivera et al. interestingly argue that hospitals with routine CTP utilization (CTP-H) do not offer a thrombectomy outcome advantage compared with hospitals where CTP use was optional (CTP-L).^33^ In the study, patients admitted to CTP-H sites were almost half as likely to receive thrombectomy compared with patients at CTP-L sites (OR = 0.59; 0.41–0.85). Despite this, outcomes and complications at CTP-L hospitals were comparable with patients from CTP-H hospitals, with patients admitted to CTP-L sites reporting better outcomes at discharge, suggesting that the implementation of perfusion imaging may potentially exclude patients from thrombectomy procedures without offering noticeable advantages. Similarly, De Muynck et al. (2020) demonstrated no significance in patient outcomes using CTP in their single-center retrospective analysis of patients with major vessel occlusions.^34^ One systematic review in 2017 evaluated 994 patients who were evaluated with perfusion-guided therapy and compared them to 1819 patients who did not. After comparing 13 studies, the analysis found that 51.1% of patients treated with perfusion imaging assistance had a non-significant favorable outcome at 3 months compared with the 45.6% of patients without perfusion imaging.^35^

Although we did not evaluate the usage of telestroke in the included studies, its implementation might have acted as a confounding variable potentially impacting CTP procedures and critical times. There exist conflicting results in the literature with regard to telestroke effects on clinical outcomes and patient selection for revascularization surgery. Some studies have shown a significant decrease in critical times when implementing telemedicine in patient evaluation.^36^ On the contrary, a recent meta-analysis showed no significant variations between the usage of telestroke and conventional treatment methods.^37^

An important factor to consider when implementing CTP imaging modalities is the financial implications of introducing and maintaining this modality. Studies in literature have supported CTP by demonstrating its cost-effectiveness in patient selection for thrombolysis. Van Voorst et al. (2023) evaluated 701 patients undergoing NCCT + CTA + CTP and compared them to a generated population receiving conventional imaging (NCCT + CTA) using a Markov model.^38^ The study determined that using CTP resulted in health gain and cost savings in AIS patients. The need for further research of patient outcome assessments is crucial before a firm conclusion can be drawn of the usefulness of CTP routine implementation at stroke centers.

### Limitations

There are some limitations to this systematic review and meta-analysis. There are no studies that have directly compared the CT + CTA versus CT + CTA + CTP. The specific mode of transportation for each stroke patient was not identified in any of the studies. The differential effect of being transported via the mothership versus drip-n-ship models may lead to differences in critical times to treatment and hence outcomes. The time when treatment was administered varied by each patient in each study, impacting the usefulness of CTP. In addition, retrospective studies showed significantly divergent results compared with their prospective counterparts when outcomes such as sICH were evaluated. These higher bleeding rates may be related to a potential selection bias that is commonly prevalent in retrospective studies. Moreover, some studies used other types of imaging in addition to CTP for the imaging selection, which may cause bias. Imaging techniques included MRI and magnetic resonance angiography (MRA)^10^ in a few of these studies. It must also be noted that not all the studies used included representative samples, as only a select few were randomized control trials. As such, applying the results to a larger population becomes more difficult with the increased chance of bias in non-randomized studies. Finally, the primary focus of our meta-analysis was on the usefulness of CTP imaging on EVT outcomes in acute stroke. CTP imaging is also increasingly being used for thrombolysis decisions in the late time window.^10^ The imaging information leads to better patient selection and lower risk of complications including sICH. Nevertheless, criteria from the National Institute of Neurologic Disorder and Stroke (NINDS) study are encouraged to be used to determine intravenous thrombolytics eligibility.^39,40^

## Conclusion

The integration of CTP-guided therapy is a useful adjunct for selecting EVT in the late window. Our findings demonstrate that the use of CTP is associated with reduced mortality rates and increases the chance of successful recanalization, offering insight to improve patient clinical outcomes. Further research is required to establish a clearer understanding of the potential advantages or limitations of incorporating CTP into stroke imaging protocols.
